# A Randomized Crossover Design to Assess Learning Impact and Student Preference for Active and Passive Online Learning Modules

**DOI:** 10.1007/s40670-015-0224-5

**Published:** 2015-12-21

**Authors:** Amy J. Prunuske, Lisa Henn, Ann M. Brearley, Jacob Prunuske

**Affiliations:** Department of Biomedical Sciences, University of Minnesota Medical School Duluth, 1035 University Dr, #SMED 321, Duluth, MN 55812-3031 USA; Division of Biostatistics, University of Minnesota, Minneapolis, MN USA; Biostatistical Design and Analysis Center and an Instructor in the Division of Biostatistics, University of Minnesota, Minneapolis, MN USA; Department of Family Medicine and Community Health, University of Minnesota Medical School Duluth, Duluth, MN USA

**Keywords:** Online, Elearning, Active learning, Lecture, Medical education, Pre-clinical

## Abstract

Medical education increasingly involves online learning experiences to facilitate the standardization of curriculum across time and space. In class, delivering material by lecture is less effective at promoting student learning than engaging students in active learning experience and it is unclear whether this difference also exists online. We sought to evaluate medical student preferences for online lecture or online active learning formats and the impact of format on short- and long-term learning gains. Students participated online in either lecture or constructivist learning activities in a first year neurologic sciences course at a US medical school. In 2012, students selected which format to complete and in 2013, students were randomly assigned in a crossover fashion to the modules. In the first iteration, students strongly preferred the lecture modules and valued being told “what they need to know” rather than figuring it out independently. In the crossover iteration, learning gains and knowledge retention were found to be equivalent regardless of format, and students uniformly demonstrated a strong preference for the lecture format, which also on average took less time to complete. When given a choice for online modules, students prefer passive lecture rather than completing constructivist activities, and in the time-limited environment of medical school, this choice results in similar performance on multiple-choice examinations with less time invested. Instructors need to look more carefully at whether assessments and learning strategies are helping students to obtain self-directed learning skills and to consider strategies to help students learn to value active learning in an online environment.

**Electronic supplementary material:**

The online version of this article (doi:10.1007/s40670-015-0224-5) contains supplementary material, which is available to authorized users.

## Background

The use of online resources and curricula by administration, educators, and learners is expanding. Online learning opportunities facilitate the distribution across institutions of a standardized, expert-driven curriculum [1, 2]. In addition, online modules can allow students to drive the learning process by determining what, when, where, and how information is accessed [3–5]. Several randomized controlled trials have shown online modules have comparable efficacy to in-class learning experiences, but due to the lack of human contact, there can be a decrease in student engagement and confidence in their understanding of the material [6–12]. Some instructors address this pedagogical challenge by adopting a blended or hybrid model where students use online lectures as preparation for an in-class active learning experience, which facilitates interaction with the instructor [13, 14]. However, this model is time-intensive and evidence supports that the benefits of the flipped classroom come from the in-class active learning component [15]. The knowledge of what online structures contribute to effective learning is limited and there is variability in how learning principles are applied to online module development [16].

Given the breadth and depth of medical school curricular content, online modules must be implemented in a way that optimizes learning using solid educational pedagogy. There is overwhelming evidence based on meta-analyses that in the classroom, student engagement in student-centered learning activities rather than passively listening to lectures results in improved performance and may be particularly important for students from underrepresented groups [17]. Online, recorded lectures allow students to learn the information in a self-paced manner, but like in-class lecture do not require students to actively engage in the material, to utilize metacognitive strategies, or to practice applying the information [18]. The development of more complex online modules including simulations, virtual laboratories, or interactive case presentations is time- and resource-intensive [19]. In addition, students’ attitudes toward the modules in the context of the other medical school curricular elements will impact their learning experience [20, 21].

Little information exists directly comparing online passive to online active learning formats on medical student exam performance or preference [22]. We provided students with online experiences using both lecture and active learning formats. The constructivist learning activity was designed with a technical scaffolding approach such that students were asked to answer a series of questions using a finite number of resources [23, 24]. At times, we gave students the option to choose between formats and in other cases we used a randomized, crossover format to expose students to both module types. We compared module-learning gains, knowledge retention of the material on the course assessments, perceived value for the two formats, time spent on the modules, and associations between module performance and student demographics.

## Materials and Methods

### Institutional Context

The University of Minnesota Medical School Duluth is a 2-year regional branch campus with a mission to create rural, family physicians and American Indian physicians. There are 60 matriculates each year with some students entering delayed programs slightly altering the number of students enrolled in each course. For the two classes in this study, 45 % of the students were female and 8 % were Native American. The average MCAT for the students in the study was 28.8 ± 2.9, and the average pre-medical school GPA was 3.55 ± 0.25. Students are enrolled in an organ system-based curriculum. Each course contains core content in the basic sciences of anatomy, pathology, pharmacology, physiology, and microbiology as well as clinical science applications including physical examination and diagnostic skills. The Neurological Medicine course is a required course of the first year curriculum. During this 8-week course, students complete approximately 100 h of lecture and 100 h of additional instruction through anatomy lab, simulation, clinical experiences, and independent learning activities. The University of Minnesota Institutional Review Board approved this study on 9/29/2011, study #1109E04785.

### Online Learning Modules

During the first iteration in 2012, a clinician and basic scientist developed seven interdisciplinary modules for the Neurological Medicine course with additional support from various content experts. Each neurological independent learning module online (NILMO) was designed to cover core content in several disciplines including headache, stroke, and seizure. The module was the primary mode of transmitting the content to the students. Prior to each module, students completed a five-question multiple-choice pre-test on the module content. The pre-test questions consisted of two-step board style questions requiring the development of higher level Bloom’s skills including analysis, synthesis, and/or application of concepts, not simple factual recall [25]. Students then chose between the two learning formats designed to address the learning objectives of the module. The first option was a 30–40-min PowerPoint lecture with an expert audio commentary. The second option was a scaffolded worksheet where students were provided a list of questions and of recommended resources. The questions asked students to reflect on short video clips, draw pictures, interact with an online eye simulator, or construct tables (Electronic Supplementary Material 1). After reviewing the module, students completed a post-test evaluation, consisting of the same five questions presented in the pre-test. Students then had the option to be done or to complete the other learning format and repeat the post-test. The final post-test evaluation score counted toward each student’s final course grade and the material was tested on the course final but not on the block exams. The NILMOs were delivered to students using an institutionally developed web-based curriculum management system regularly accessed by the students.

**Fig. 1:**
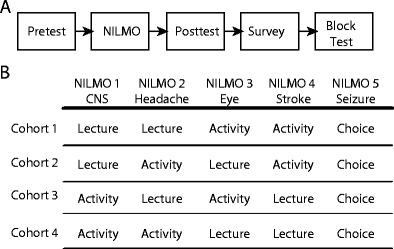
Overview of the online interventions. **a** Prior to the module, students completed a five-question pre-test, and after completing the module, students completed a post-test and survey. Student performance on the module learning objectives was monitored on the course block tests and on the course final exam. **b** Students were randomly assigned into four cohorts and assigned to complete two of the lecture NILMOs and two of the activity NILMOs. For the fifth NILMO, students chose which format to complete. The topics associated with the NILMOs are shown below the name with NILMO 1 being an overview of the central nervous system (CNS)

During the second iteration in 2013, the course was reorganized into five blocks that included five of the previous seven modules. Rather than giving students the choice between the lecture and activity formats, we randomized students into four cohorts and assigned in a crossover fashion, each cohort to complete two lectures and two activities (Fig. 1). For the fifth module, students were given the option to choose between the lecture and the activity. In the previous year, students received delayed feedback from the instructors on their answers to the assignment questions, so we integrated an immediate feedback system to support learning [26]. After submitting their answer to an activity question, students unlocked an expert answer and a subset of their peers’ answers. For each module, students completed the same five-question pre-test and post-test assessments and a survey as to the value of the experience and the amount of time they spent on the module. In this iteration, the post-test evaluations were not counted toward total course points; however, questions relating to the NILMO content were included on the five-block tests, and if students did not complete the module, they were ineligible to receive the associated NILMO points on the block test.

### Data Analysis

Student performance on pre- and post-tests at the beginning and end of each module was used to measure learning gains. Between three and six questions on each of the block tests directly related to the modules were used to evaluate short-term knowledge retention. Finally, seven questions on the final exam were used to evaluate long-term knowledge retention. We assessed students’ format preferences using descriptive statistics and by reviewing narrative survey feedback. Immediately after completing each module, students’ self-reported time spent on the module as 0–30 min, 31 min–1 h, 1–2 h, 2–3 h, 3–4 h, 4–5 h, and more than 5 h, and perceived value as useless, some benefit, significant, or essential. Students provided free text additional comments at the end of the module and end of the course.

Data analysis was carried out in SAS 9.2 and SAS 9.3 (SAS Institute Inc., Cary, NC, USA). We used linear regression to analyze for associations between overall performance on the NILMOs (total points) and the course final exam score and for associations between performance on each NILMO and the associated block exam. We used two sample *t* tests and cumulative logistic and repeated measures model fitting as appropriate. We analyzed for univariate factors associated with learning gains on each of the models using the non-parametric Kruskal-Wallis test, Fisher’s exact test, and Spearman’s rank correlation tests. Multiple linear regression models were used to determine whether there was an association between the NILMO block score and possible predictors of performance including activity choice, minority status, gender, MCAT (total score and MCAT subscores in verbal reasoning, physical sciences, biological sciences, and writing sample), undergraduate GPA (total and biosciences subscore), and time spent on the module. Multivariate modeling was done to explore possible conditional associations between the final NILMO score and the same set of suggested predictors.

## Results

### Student Preferences

In the 2012 iteration, we sought to compare online lecture and online active formats by giving students a choice to either view an online lecture or complete a structured worksheet. The modules were developed for a neurological medicine course and contained core content that was primarily presented in the module. The lecture component consisted of a recorded PowerPoint presentation, and the scaffolded activity required students to answer a series of questions using recommended resources. Students had the option to complete both formats and earned points toward their course grade for completing either format. All of the students completed the modules, but students overwhelmingly selected to complete only the lecture option, which prevented a comparison of learning gain by format in this iteration. Ten students selected to complete an activity only 16 times out of the 441 possible selection opportunities (3.6 %) and 11 of those times, a student chose to complete both the lecture and the activity. The narrative data collected from the students suggested an increased level of familiarity and comfort with the lecture format of learning, which is more consistent with most of their prior medical school coursework.

Therefore, in the second year, we utilized a randomized crossover design to expose students to both learning formats and we decreased the number of modules to 5 (Fig. 1b). Students were randomly assigned to complete two lecture and two activity modules, and for the final module, students were given a choice between the formats. All of the students completed the modules, and even after being exposed to both module types, students still overwhelmingly chose the lecture option with only 1 out of 58 students choosing to complete the activity for the final module.

### Student Performance by Format

Even though the students strongly preferred the lecture format, it is possible that the students doing the activity would show higher performance than the students who watched the lecture. The randomized crossover design allowed us to look at the differences between the formats in a controlled manner. We found that both formats resulted in increased learning gain, but that there were no significant differences in gain between the two formats on any of the modules. The mean learning gain for each of the modules (post-test minus pre-test) on a five-point scale ranged from 0.52 to 2.59 (Table 1). The average student learning gain for the lecture format was 1.54 and for the active format was 1.24 points.

**Table 1:** Learning gains for randomly assigned students completing either the activity or lecture

Module	Choice	Mean	Confidence interval	Variance treatment (*p* value)
NILMO 1	Activity	2.07	(1.69, 2.45)	Pooled (0.34)
	Lecture	1.83	(1.37, 2.28)	
NILMO 2	Activity	1.32	(0.66, 1.98)	Pooled (0.10)
	Lecture	2.59	(2.11, 3.06)	
NILMO 3	Activity	0.71	(0.082, 1.35)	Pooled (0.73)
	Lecture	1.21	(0.62, 1.81)	
NILMO 4	Activity	0.86	(0.46, 1.27)	Pooled (0.24)
	Lecture	0.52	(0.013, 1.02)	

We evaluated short- and long-term retention of the module content by students’ performance on the block tests and the course final exam. Student performance on questions related to the module material was compared depending on whether the students had viewed the lecture or completed the activity. We saw no significant difference in student performance by format. In addition, there were no associations between module learning gains and performance on associated block or final exam questions or student demographics including gender, MCAT, undergraduate GPA, or race. Therefore, the use of the active modules did not appear to help a particular group of students.

### Student Perceived Value

**Fig. 2:**
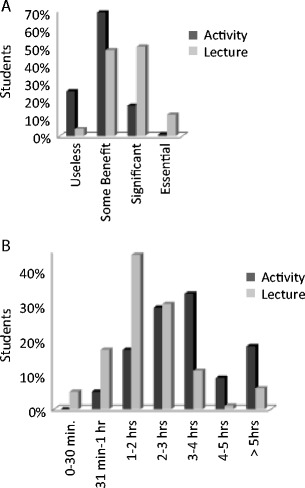
Student attitudes toward and time estimates for the completion of the online modules. **a** Immediately after completing each module, students were asked to report the value of the lecture and/or assignment as useless, some benefit, significant, or essential. **b** Students estimated the amount of time they spent completing the entire module. The percentage of students choosing each category was averaged over the five NILMOs

Given that the students demonstrated learning gains for both formats, we wanted to assess how valuable the students felt the formats were. We used student survey data collected immediately after completing the online module to compare student attitudes toward the formats. Students were more likely to rate the experience of viewing the lecture as being essential or significantly beneficial toward their mastery of the information (Fig. 2a). This is in contrast to their rating of the activity, which was more likely to be rated as useless or only having some benefit. There was no association between the student rating of the value of the activity and their module learning gain or their performance on the material on the block examination. Students were found to value the lecture more than the activity both years of the study even after the modules were tailored to provide enhanced feedback to the user.

We also collected student narrative comments at the end of the course both years to gain a better understanding of student perspectives. The student response rate at the end of course surveys was 69 %. One student said “I want to look at your lecture and be told what is important” and another said, “looking things up takes a lot of time I don’t believe it is worthwhile”. The students disliked that they were not able to ask questions during either format. One student did acknowledge, “as much as I dislike doing NILMO activities, I would say that I learn the content better than if I just listened to a lecture”.

We then looked at how time varied between the completions of the two different module formats. We found that students reported spending around 3 h on the activity module and around 2 h on the lecture module (Fig. 2b). This amount of time commitment was comparable to the amount of curricular time students spent on other core topics. A subset of students reported spending more than 5 h on either the lecture or activity. The time spent on the modules and the learning gains decreased over the modules. Note we did not find that spending more time on the module correlated with an improved performance on the post-test or on material on the course examinations.

## Discussion

In a pre-clinical course, students strongly prefer online lectures to online constructivist learning activities. Students demonstrate equivalent short-term learning gains and knowledge retention regardless of educational format. Given the rapid rate of the expansion of medical knowledge, limited resources for the development of medical curricula, and student interest in technology, online curricular innovation is likely to become an integral part of most medical education programs. Knowledge of student preferences and the relative effectiveness of various curricular formats will assist medical educators as they plan and implement online curricula.

We attempted to avoid pitfalls previously shown to decrease student satisfaction with online learning [10]. To facilitate student accessibility and ease of use, the modules were integrated into the familiar course management system and students reported few technical challenges working with the modules. Online resources are sometimes viewed as supplemental or optional activities. Students completed all of the modules in this study since this was their only exposure to these important topics and because they earned points toward their grade for the completion of the modules. Through the course management system, students had access to only one of the resources and were directed to complete the module independently using the assigned resource. In the event that cross sharing occurred, we do not believe that this meaningfully complicates our analysis since the benefit of the constructivist activity is derived from completing the activity.

We incorporated general principles that lead to more effective learning including feedback, activity, individualization, and relevance [27]. Students valued having the pre-/post-test to provide feedback on their understanding of the material, but adding the timely expert and peer answer responses did not positively impact students’ attitudes toward the activity-focused modules. This lack of engagement with the module indicates that independently, students may be less able to actively engage with the material [28]. Individualization was incorporated in that students could choose to answer questions by drawing pictures or writing explanations to the questions. Relevance can be a challenge during the first year medical curriculum so a clinician was engaged in the development of the modules. One of the modules provided a link for students to interact with an online eye simulator, but even in this case, students indicated that they would prefer to have the information communicated to them by PowerPoint.

The social structure or field of the course can impact student willingness to invest in active learning [29]. This was a very time-consuming course with students in class on average 6 h a day. Therefore, successful students adapted a disposition that valued efficiency to acquire the desired capital of course points and increased time on task for this exercise did not result in an improved performance on the examination questions. The limited time became more challenging further into the course and students’ learning gains on later modules using either format were low. One of the students in the course wrote that it is “Ironic, we’re learning about learning and memory (or we will be anyway) but are not able to do things like the assignment.” Therefore, students seem to be aware of the value of active learning but are choosing the less time-intensive path as indicated by how long they estimated spending on each of the formats. Students need to have sufficient time to meaningfully engage in online active learning. Students become less self-directed in their approach to learning over the first year of medical school [30]; thus, there is a reason to prioritize learning activities that help students develop self-directed learning skills.

Assessment drives learning. There was no difference between the formats in performance on multiple-choice assessments that required students to apply the information in the module. We had a limited number of questions on our pre/post-tests and some students scored well on the pre-test and post-test, which left little room for improvement and variability between students. We may have failed to detect a significant difference between the two formats as the difference was small and we had a small student sample size, but the active learning exercises also did not result in a significant improvement in exam performance. In the current study, we were unable to assess whether the modules enhanced self-directed learning skills or patient outcomes since these students are not regularly engaged in clinical practice. We chose to use the multiple-choice board style questions as this is the primary form of assessment in our curriculum and on the board exams, but this format may not be effective at measuring differences between the two learning formats.

Collaborative learning is an important feature that is often missing from online learning. Physicians engaged in continuing medical education generally highly rate web-based learning [10], but in the first year of medical school having the support of peers and instructors for application of course content may be necessary to develop student self-efficacy. Since 2014, the content of these modules has been delivered in person as group activities in an active learning classroom. Students are more engaged and feedback on the constructivist active modules has been positive, suggesting that this type of learning format may occur better in person than online in the first year student population. A limitation of our study is that it was implemented at a single medical school, so we cannot conclude whether these results are transferable to other first year medical school environments.

## Conclusion

Previous work has focused on comparing online to active learning environments, but given the rapid rate of expansion of medical knowledge and increasing time demands, it is important to also identify the most effective online learning formats. We found students preferred online lecture rather than completing online constructivist activities and that this choice results in similar performance on the standard medical school multiple-choice examinations with less time invested. At the same time, an important goal of medical education is to foster self-directed learning and so instructors need to reflect when developing online curriculum on what skills their assessments and learning strategies are building as well as what other curricular elements may be occupying the students’ time. Given the challenge for students in completing constructivist activities online, it will also be important to devise additional strategies to support students in this environment.

NILMO, neurological independent learning modules online

## Electronic Supplementary Material

Below is the link to the electronic supplementary material.

ESM 1 Student answer from online constructivist learning activity (PDF 1273 kb)

## References

[1] Cook DA, Levinson AJ, Garside S, Dupras DM, Erwin PJ, Montori VM (2010). Instructional design variations in internet-based learning for health professions education: a systematic review and meta-analysis. Acad Med.

[2] Day FC, Srinivasan M, Der-Martirosian C, Griffin E, Hoffman JR, Wilkes MS (2015). A comparison of Web-based and small-group palliative and end-of-life care curricula: a quasi-randomized controlled study at one institution. Acad Med.

[3] Beale EG, Tarwater PM, Lee VH (2014). A retrospective look at replacing face-to-face embryology instruction with online lectures in a human anatomy course. Anat Sci Educ.

[4] Lambert DR, Lurie SJ, Lyness JM, Ward DS (2010). Standardizing and personalizing science in medical education. Acad Med.

[5] Masters K, Gibbs T (2007). The spiral curriculum: implications for online learning. BMC Med Educ.

[6] Prunuske J (2010). Live and Web-based orientations are comparable for a required rotation. Fam Med.

[7] Jenkins S, Goel R, Morrell DS (2008). Computer-assisted instruction versus traditional lecture for medical student teaching of dermatology morphology: a randomized control trial. J Am Acad Dermatol.

[8] Wofford MM, Spickard AW, Wofford JL (2001). The computer-based lecture. J Gen Intern Med.

[9] Williams C, Aubin S, Harkin P, Cottrell D (2001). A randomized, controlled, single-blind trial of teaching provided by a computer-based multimedia package versus lecture. Med Educ.

[10] Chumley-Jones HS, Dobbie A, Alford CL (2002). Web-based learning: sound educational method or hype? A review of the evaluation literature. Acad Med.

[11] Solomon DJ, Ferenchick GS, Laird-Fick HS, Kavanaugh K (2004). A randomized trial comparing digital and live lecture formats [ISRCTN40455708. BMC Med Educ.

[12] Wiecha JM, Chetty VK, Pollard T, Shaw PF (2006). Web-based versus face-to-face learning of diabetes management: the results of a comparative trial of educational methods. Fam Med.

[13] Prunuske AJ, Batzli J, Howell E, Miller S (2012). Using online lectures to make time for active learning. Genetics.

[14] McLaughlin JE, Roth MT, Glatt DM (2014). The flipped classroom: a course redesign to foster learning and engagement in a health professions school. Acad Med.

[15] Jensen JL, Kummer TA, Godoy PDdM (2015). Improvements from a flipped classroom may simply be the fruits of active learning. CBE Life Sci Educ.

[16] Lau KH (2014). Computer-based teaching module design: principles derived from learning theories. Med Educ.

[17] Freeman S, Eddy SL, McDonough M (2014). Active learning increases student performance in science, engineering, and mathematics. Proc Natl Acad Sci U S A.

[18] Bransford J, Brown A, Cocking R (2015). How people learn. Brain, mind, experience, and school Washington, DC.

[19] Polly P, Marcus N, Maguire D, Belinson Z, Velan GM (2014). Evaluation of an adaptive virtual laboratory environment using western blotting for diagnosis of disease. BMC Med Educ.

[20] Longmuir KJ (2014). Interactive computer-assisted instruction in acid-base physiology for mobile computer platforms. Adv Physiol Educ.

[21] Palmer E, Devitt P (2014). The assessment of a structured online formative assessment program: a randomised controlled trial. BMC Med Educ.

[22] Cook DA, Levinson AJ, Garside S, Dupras DM, Erwin PJ, Montori VM (2008). Internet-based learning in the health professions: a meta-analysis. JAMA.

[23] Yelland N, Masters J (2007). Rethinking scaffolding in the information age. Comput Educ.

[24] Brandon AF, All AC (2010). Constructivism theory analysis and application to curricula. Nurs Educ Perspect.

[25] Crowe A, Dirks C, Wenderoth MP (2008). Biology in bloom: implementing Bloom’s taxonomy to enhance student learning in biology. CBE Life Sci Educ.

[26] Cook DA, Levinson AJ, Garside S (2010). Time and learning efficiency in internet-based learning: a systematic review and meta-analysis. Adv Health Sci Educ Theory Pract.

[27] Harden RM, Laidlaw JM (2013). Be FAIR to students: four principles that lead to more effective learning. Med Teach.

[28] Andrews TM, Leonard MJ, Colgrove CA, Kalinowski ST (2011). Active learning not associated with student learning in a random sample of college biology courses. CBE Life Sci Educ.

[29] Bourdieu P (1977). Outline of a theory of practice.

[30] Premkumar K, Pahwa P, Banerjee A, Baptiste K, Bhatt H, Lim HJ (2013). Does medical training promote or deter self-directed learning? A longitudinal mixed-methods study. Acad Med.

