# Supplementary material for: A Randomized Crossover Design to Assess Learning Impact and Student Preference for Active and Passive Online Learning Modules
Source: Med Sci Educ. 2015 Dec 21;26:135–41. doi: 10.1007/s40670-015-0224-5 (PMC4819804; doi:10.1007/s40670-015-0224-5)

Prunuske et al.

Additional file 1: Student answer from online constructivist learning activity

Students were asked to draw a picture of the serotonergic neuron and synapse found in the brain as part of the headache module (NILMO2). Students were directed to include all sites of action of serotonin, to draw the mechanisms of action for serotonin and include relevant enzymes, and to draw the sites of action and role of DHE, Triptans, and amitriptyline.

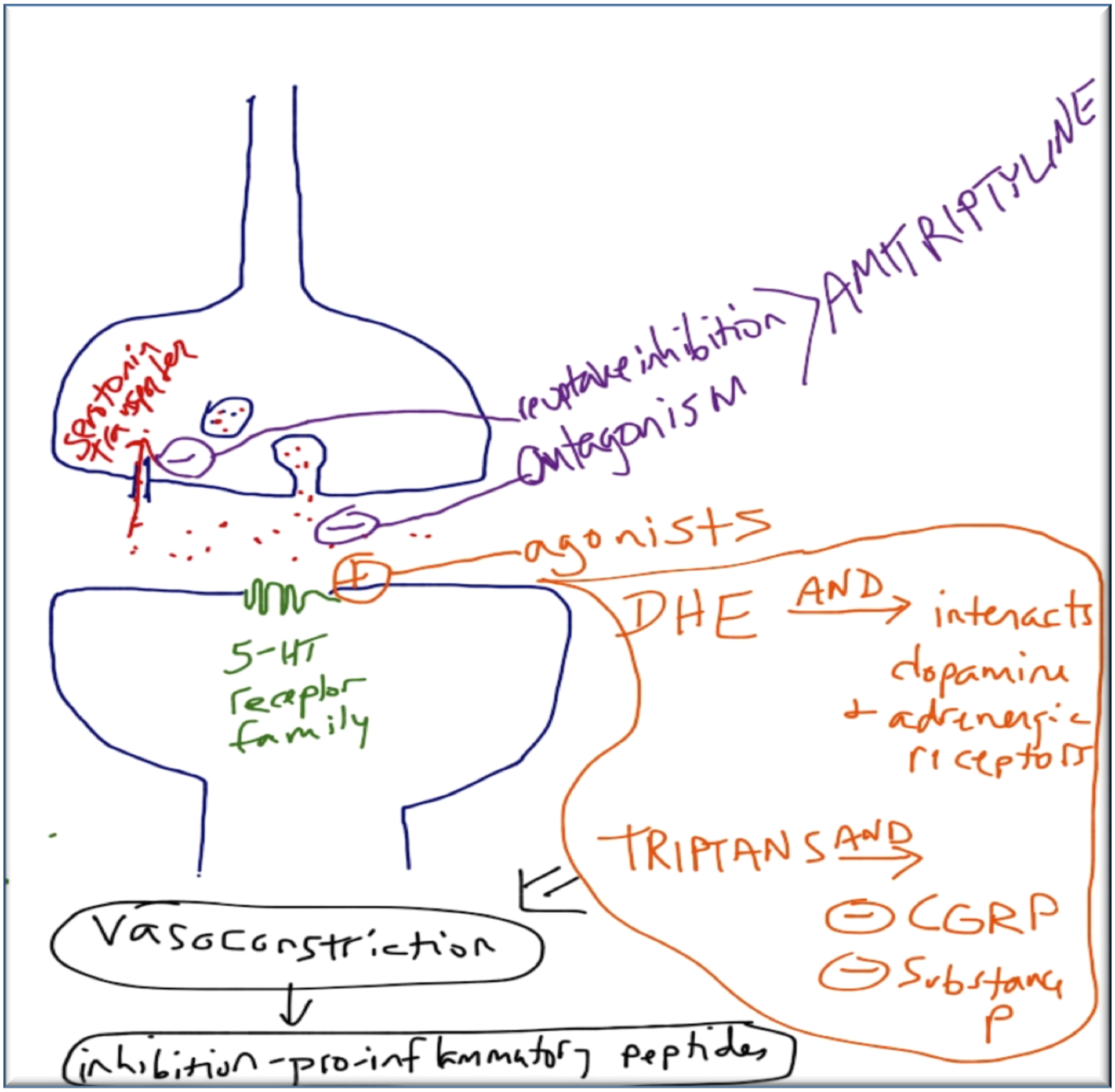

Supplement: Supplementary file 1 — Student answer from online constructivist learning activity (PDF 1273 kb) [file 40670_2015_224_MOESM1_ESM.pdf]
